# Evidences of emerging pain consciousness during prenatal development: a narrative review

**DOI:** 10.1007/s10072-022-05968-2

**Published:** 2022-03-04

**Authors:** Raffaele Falsaperla, Ausilia Desiree Collotta, Michela Spatuzza, Maria Familiari, Giovanna Vitaliti, Martino Ruggieri

**Affiliations:** 1 Neonatal Intensive Care Unit and Neonatal Accompaniment Unit, Azienda Ospedaliero-Universitaria Policlinico “Rodolico-San Marco,” San Marco Hospital, University of Catania, Catania, Italy; 2 Unit of Clinical PaediatricsAzienda Ospedaliero-Universitaria Policlinico“Rodolico-San Marco”, San Marco Hospital, Catania, Italy; 3 Postgraduate Training Program in Pediatrics, Department of Clinical and Experimental Medicine, University of Catania, Catania, Italy; 4 Institute for Biomedical Research and Innovation - The National Research Council of Italy (IRIB-CNR), Catania, Italy; 5 Unit of Pediatrics, Department of Medical Sciences, Section of Pediatrics, University Hospital Sant’Anna, University of Ferrara, Ferrara, Italy; 6 Unit of Rare Diseases of the Nervous System in Childhood, Department of Clinical and Experimental Medicine, Section of Pediatrics and Child Neuropsychiatry, University of Catania, AOU “Policlinico,” PO “G. Rodolico,” Via S. Sofia, 87, 95128 Catania, Italy

**Keywords:** Onset of consciousness, Fetal life, Preterm neonate, Newborn

## Abstract

**Background:**

The study of consciousness has always been considered a challenge for neonatologists, even more when considering the uterine period. Our review aimed to individuate at what gestational age the fetus, which later became a premature infant, can feel the perception of external stimuli. Therefore, the aim of our review was to study the onset of consciousness during the fetal life.

**Materials and methods:**

A literature search was performed in Medline-PubMed database. We included all papers found with the following MeSH words: “consciousness or cognition or awareness or comprehension or cognitive or consciousness of pain” in combination with “embryo or fetus or fetal life or newborn.” Studies were selected if titles and/or abstracts suggested an association between formation of consciousness (the basics of neurodevelopment) and preterm infant or fetus. Titles and abstracts were first screened by three independent reviewers according to Cochrane Collaboration’s recommendations.

**Results:**

From the literature review, we found only 8 papers describing the onset of consciousness in the transition period from fetus to premature newborn. Therefore, according to these papers, we temporally analyzed the formation of the thalamocortical connections that are the basis of consciousness.

**Conclusions:**

We can conclude that from a neuroanatomical point of view, it is rather unlikely that the infant can be seen as a conscious human before 24 weeks of gestational age, thus before all the thalamocortical connections are established. Further literature data have to confirm this hypothesis.

## Introduction

Consciousness identifies a state in which a patient is awake, alert, responsive to stimuli, and aware of him/herself and the outside world [1, 2]. The capacity to be conscious can be considered the crucial sign of human existence [1, 2]. The topic is important above all when clinicians have to afford those patients under life-sustaining therapy. In this regard, it is questionable whether to continue a life-sustaining therapy when consciousness is not yet fulfilled or not in those fetuses needing this medical life support [1, 2] or when it should be suspended.

By definition, phenomenal consciousness involves an idealized and hypothetical situation of pure subjective experience (usually called “qualia”) without further associated information processing (and, consequently, no need for verbal communication). Access consciousness refers to the fact that conscious information, unlike the unconscious one, is accessible to numerous cognitive processors, such as those mediating working memory, motor behavior, or verbal report. The importance of this distinction remains deeply debated, but it has been suggested that “global availability of information (…) *is* what we subjectively experience as a conscious state” [1]. In regard to the “global neuronal workspace” (GNW) theory, it has been proposed to explain the construction of consciousness [2], according to which perceptual contents, which are acted upon by localized processors, only become conscious when they are widely broadcasted to other processors across the brain. This process of broadcasting implies that the information in the workspace becomes available to many local processors, and it is the ample accessibility of this information that is hypothesized to constitute conscious experience. The theory, firstly described by Baars, involves processors related to different timings, including the past (memory), the present (sensory input and attention), and the future (motor plans, verbal report, and value systems) [2]. Thus, the GNW achieves experiential integration in terms drawn from the philosophy sphere of mind, both synchronic (at a particular point) and diachronic (over time). The author proposed the diffuse, extended reticular-thalamic activating system as the main brain structure involved in the formation of the GNW. Nevertheless, the initial hypothesis of Baars did not distinguish between the level of conscious processing (under the reticular formation control) and the content. By contrast, Dehaene et al. later proposed a defined brain network as the neural instantiation [3]. In addition to specialized, localized, and modular cortical areas that process specific motor, perceptual, evaluative, and memory information, a second computational space should be composed of widely distributed excitatory neurons (named GNW neurons), with long age axons, constituting reciprocally connected tracts that through descending connections are able to “selectively suppress or mobilize the contribution of specific processor neurons.” This distributed population of neurons seems to possess the ability to transmit top-down information and to receive bottom-up information from all the other processors within the brain, thus selecting broadcast information [3]. At a neuronal level, the GNW hypothesis suggests a key role for large pyramidal cells in the II and III cortical layers, but also the contribution of pyramidal cells in deeper cortical layer V. In this context, human consciousness, thinking, and emotions are assumed to be the product of the activity of the cerebral cortex and the brainstem [1, 2].

The full-term newborn shows some signs of consciousness such as being awake and aware of him/herself and the mother. Infants at this age express primary emotions such as joy, disgust, and surprise, and they usually remember rhymes and vowels to which he or she has been exposed during fetal life [1].

There is little information in the literature about consciousness during fetal life or in premature infants. The few reviews address issues on pain perception or neonatal consciousness including expert opinions and other literature data from extensive reviews [1–4]. No scientific evidences have been published in this regard.

In light of the emerging evidences, the aim of our review is to study consciousness in fetal life, trying to focus on the exact time consciousness is settled and to consider from which gestational age vital-life support should be continued or interrupted, and in which extent.

## Materials and methods

A systematic search of the PubMed MEDLINE and Embase database was performed, according to Preferred Reporting Items for Systematic Reviews and Meta-Analyses (PRISMA statement), using the following MeSH words: “consciousness or cognition or awareness or comprehension or cognitive or consciousness of pain” in combination with “embryo or fetus or fetal life or newborn”.

The filters we used were restricted to neonates, fetus or embryo fetus, human studies, and English language.

References to articles that were recovered using this search strategy were also examined to retrieve further relevant publications.

The inclusion criteria were (i) all papers describing the genesis of consciousness in relation to gestational age and (ii) studies reporting a population with gestation age less than 40 weeks.

The exclusion criteria were (i) all works that did not address the genesis of consciousness from the temporal point of view in relation to gestational age; (ii) all works that included a population under examination over 40 weeks of gestation; and (iii) reviews, systematic reviews, guidelines, survey, meta-analysis, editorial letters, expert opinions, and case reports reporting only theoretical point of view on the topic, without scientific basis on human samples.

The search was performed without date limitations and was current until June 15, 2021.

Studies were selected if the title and/or abstract suggested an association between formation of consciousness (the basics of neurodevelopment) and fetal age or neonatal period with a gestational age less than 40 weeks.

Titles and abstracts were first screened by three independent reviewers according to Cochrane Collaboration’s recommendations (Higgins J and Green S., 2011). All full texts were read by the same authors, and the data was extracted and organized in Excel table (Microsoft Corp.) and discussed within the group to assess quality indicators and reliability.

## Results

A total of *N* 22,429 titles were screened in PubMed and Embase after a first research analysis using the abovementioned MeSH words. After a first search, 543 papers were excluded for records because of duplicates; 232 for records marked as ineligible by automation tools; and 342 records were removed because non-human studies or in English language.

**Fig. 1:**
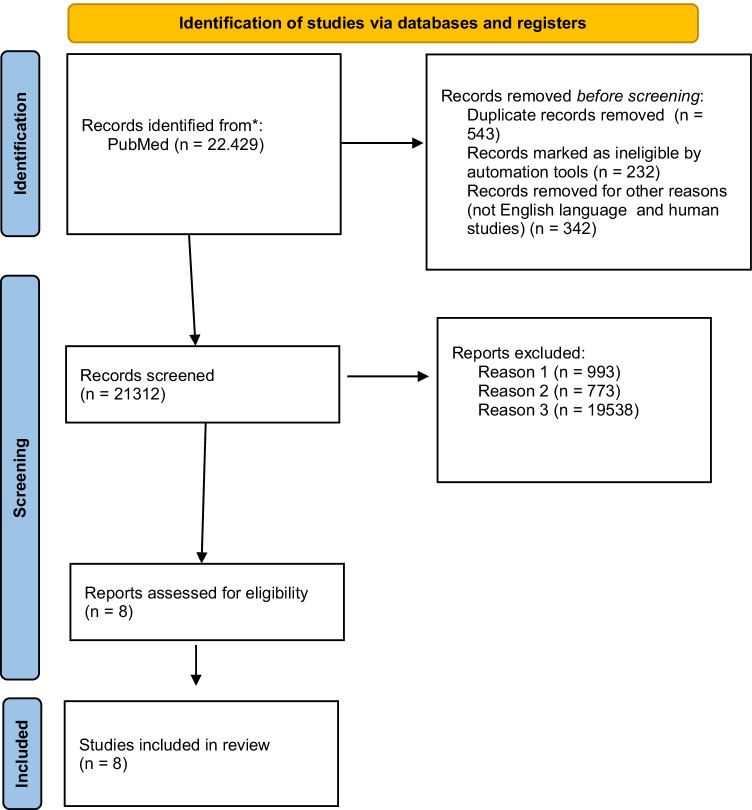
PRISMA 2020 flow diagram for new systematic reviews which included searches of databases and registers only. From Page et al. [56]. For more information, visit http://www.prisma-statement.org/

**Fig. 2:**
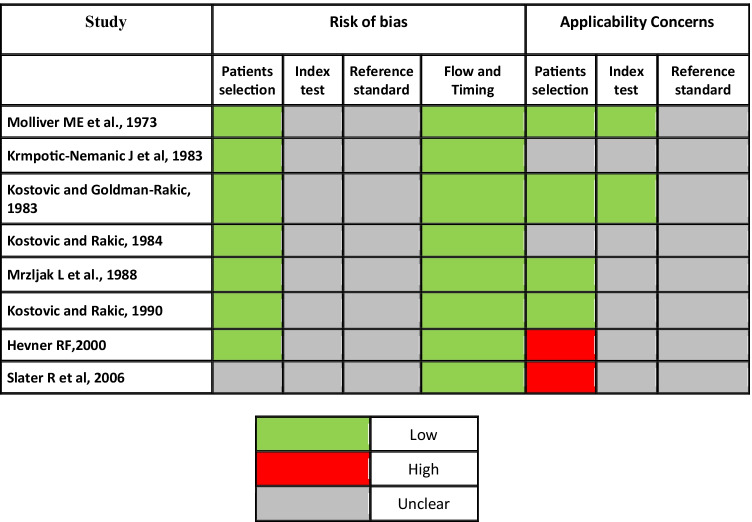
Quality assessment of included studies using QUADAS-2 tool

After this first search, *n* 21,312 abstracts were read. Among these papers, 993 were excluded because they did not mention the genesis of consciousness from the temporal point of view in relation to gestational age (Fig. 1, Reason 1); 773 were excluded because they were studies performed on patients with gestational age over 40 weeks (Fig. 1, Reason 2); and 19,538 because they were reviews, systematic reviews, guidelines, survey, meta-analysis, editorial letters, expert opinions, and case report, excluded because they did not properly focus on the formation of consciousness in fetal age or in neonates under 40 weeks of gestation (Fig. 1, Reason 3). Therefore, finally, we included *n* 8 articles that matched with our MeSH words and with our inclusion and exclusion criteria Fig. 2.

In our systematic revision, we examined exclusively case reports and clinical trials performed in fetal life or in premature infants. The exclusion criteria were based on the fact that we focused on the formation of consciousness in extremely premature infants, in order to set up a correct pain therapy. In fact, when newborns are properly conscious, they perceive and feel pain for all maneuvers required to perform resuscitation and vital supply. In this regard, we should consider that the assistance of extremely premature babies, in whom consciousness circuits are not properly formed, should be different from the assistance of premature infants with proper neurological connections that allow the baby to be aware of himself and the surrounding environments.

Therefore, in total, 8 articles were screened (Table 1, Table 2). Among these, only one study evaluated the premature infant through the real-time near-infrared spectroscopy [5], while 7/8 studies were performed on therapeutically aborted fetuses [6–12] through immunohistochemical techniques or electron microscopic analysis. Two of these seven papers also studied the brains of premature infants which passed away because of non-brain causes (e.g., sarcoma and pneumonia) a few hours after birth [9, 12].

**Table 1:** Summary of retrieved evidence (alphabetical order by first author)

Articles, year of publication	Age (gestational weeks)	Type of population examined	Study modalities
Hevner RF,2000	20–22	Therapeutically aborted fetuses	Injected with the fluorescent tracer DiI in the brain of aborted fetuses
Kostovic and Goldman-Rakic, 1983	10–30	Aborted fetuses	Histochemical analysis using acetylthiocholine iodide and Nissl methods
Kostovic and Rakic, 1984	10–30	Aborted fetuses	Nissl staining and acetylcholinesterase histochemistry
Kostovic and Rakic, 1990	8–28 26–36	Aborted fetuses and premature infants	Electron microscopic analysis
Krmpotic-Nemanic J et al., 1983	10–28	Aborted fetuses	Acetylcholinesterase histochemistry
Molliver ME et al., 1973	8–24	Aborted fetuses	An electron microscopic method for analyzing synapse locations was improved by utilizing a digital display of the *x*–*y* coordinates of each area of the specimen being inspected
Mrzljak L et al., 1988	10–34	Aborted fetuses and premature infants died of sarcoma and pneumonia	Histological examination (Golgi-Stensaas and rapid-Golgi staining techniques)
Slater R et al., 2006	25–45	Premature infants	The changes in cerebral oxygenation over the somatosensory cortex were measured in response to noxious stimulation using real-time near-infrared spectroscopy

**Table 2:** The formation of brain connections at the basis of consciousness from a temporal point of view

Anatomical/functional characteristic	Description	Gestational age	*Works*
Thalamic afferents	Thalamic afferents reach subplate zone	18–26	*Mrzljak L *et al*., 1988*
		20–22	*Kostovic and Rakic, 1990*
	Thalamic afferents reach cortical plate	23–24	*Kostovic and Goldman-Rakic, 1983* *Kostovic and Rakic, 1984* *Molliver ME *et al*., 1973*
		27–35	*Mrzljak L *et al*., 1988*
	Thalamic afferents reach visual cortical plate	23–27	*Kostovic and Rakic, 1984*
		23	*Hevner, 2000*
	Thalamic afferents reach auditory cortical plate	24–28	*Krmpotic-Nemanic *et al*., 1983*
Cortical function	Somatosensory cortical activation in response to peripheral noxious stimulation	25–28	*Slater R *et al*., 2006*

In regard to gestational age, 1/8 of these studies was achieved on infants aged between 25 and 4–5 weeks of gestational age (GW) [5], while 7/8 of these studies were performed on fetus less than or equal to 28 GW [6–12].

### Quality assessment

All included studies were assessed using QUADAS-2 (Quality Assessment of Diagnostic Accuracy Studies), a tool to assess the quality of primary diagnostic accuracy studies included in systematic reviews focused on risk of bias and applicability in the study.

Judgment regarding risk of bias is to be based on the predefined signaling questions with regard to the following four domains: patient selection, index test, reference standard, and flow-timing.

Judgment regarding applicability is based on the extent to which bias in any domain is likely to affect the question in the review.

The risks of bias and applicability concerns were rated as “low,” “high,” or “unclear.”

Unfortunately, we cannot answer the questions of index test, reference standard on risk of bias, and reference standards on applicability because there is no reference standard.

The same thing concerns the flow and timing of the risk of bias, but unlike the others, we can answer positively to one of the 3 questions, namely that all the patients enrolled were included in the analysis.

Three articles deal with the whole genesis of the brain from the neurons of the subcortex and cortex to the connections between the thalamus and the cortex [5, 9, 12].

Other 3 works instead focus on the development of the auditory and visual cortices [6, 8, 10].

Therefore, 6/8 articles deviate a little from our RS.

### Results of quality assessment

**Fig. 3:**
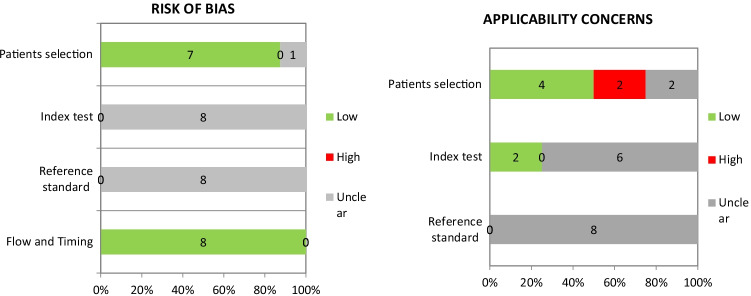
Percentage of the studies with risk of bias and applicability concerns in different domains of QUADAS-2 tool

The results of quality assessment using QUADAS-2 tool are summarized in Fig. 2, and proportions of studies with risk of bias and applicability concerns are graphically displayed in Fig. 3.

There was generally a low risk of bias regarding “flow-timing” and “index test.”

Unfortunately, there is an intermediate risk for index test, reference standard on risk of bias, and reference standards on applicability because there is no reference standard and so we cannot answer the questions of this domains.

Two of the 8 articles deal with the whole genesis of the brain from the neurons of the subcortex and cortex to the connections between the thalamus and the cortex [9, 12].

One of the 8 articles develops on understanding the cortical response to pain in human infants [5].

Other 3 instead focus on the development of the auditory and visual cortices [6, 8, 10].

Therefore, 6/8 articles deviate a little from our RS; for this reason, they have an intermediate risk for index test on applicability.

## Discussion

The study of consciousness has always been considered a challenge for neonatologists and gynecologists. However, by the introduction of new brain imaging techniques, particularly functional magnetic resonance (fMRI) and near-infrared spectroscopy (NIRS), it is now possible to assess the processing of sensory input in the cerebral cortex even on fetus.

It is even more difficult to study consciousness in the uterine period. In fact, the only existing studies in the literature are bioptic researches on the brain of aborted fetuses [6–12].

One indirect method to establish the onset of consciousness is the study of painful responses. In the fetus and in neonatal age, consciousness is formed by archaic responses, as at this age no life experience has been recorded so far to acquire emotional consciousness. In this regard, the pain is one of the most ancient responses, connected to the first stadium of consciousness. The postnatal development of painful responses and pain processing in both animals and humans has been the topic of considerable research in these last years [13]. Nevertheless, we have to mention that all the listed methods are indirect as the fact that the sensory input can reach the cortex is at best a permissive condition for consciousness but not a demonstration of an actual conscious state.

Different measures (behavioral, physiological, and biochemical studies) [14, 15] have shown that robust nociception can be also present in the youngest infants, as they react to both hypersensitivity and noxious stimulation after superficial and deep-tissue injuries as shown by grimacing [16], and spinal withdrawal reflexes [17, 18]. However, poor literature data is present on the development of human pain processing and conscious responses.

The robust response to noxious stimuli in preterm babies is supposed to be mediated at spinal or brainstem levels [19], with poor cortical involvement. Indeed, in neonates under 32 weeks of gestation, behavioral and autonomic responses are similar both in normal infants and in babies with brain injuries within the white matter [20]. It is therefore suggestible that despite anatomical evidences of thalamocortical projections in the neonatal brain from 24 weeks of gestation [3], functional nociceptive networks composed by cortical cells and intracerebral circuits have not developed until much later. The triple withdrawal reaction to noxious stimulus is certainly a reflex response integrated at spinal level and also grimaces may be considered automatic mechanisms not related to any conscious pain perception.

Considering that the experience of pain is constituted by affective and emotional components, requiring a higher level of cortical brain processing, studies on the maturation of the fetal brain development and the newborn cortical responses to painful stimuli would be helpful in understanding the infant pain experience and the onset of consciousness as consequence. In this regard, literature data have been published on somatosensory-evoked potentials in infants from 27 weeks of gestational age [21, 22], focusing mainly on the study of somatosensory pathways and prognostic indexes of neurological outcome and disease.

Neuroimaging methods have been performed to analyze the cortical pain processing in adulthood, but they cannot be applied to neonates in neonatal intensive care units (NICU). Interestingly, by using NIRS, spontaneous neuronal activity has been detected in somatosensory areas in preterm infants when they have reached term age, and it has also been detected in non-sedated healthy full-term infants [5, 23]. The sensorial information is transmitted within the preterm infant cortex from the age of 25 weeks of gestation [5].

This hypothesis was assumed by the suggestion that increasing hemodynamic responses, as diagnosed by NIRS, should correlate with increasing cortical activity [5]. The authors also found that the smaller cortical responses to painful stimuli in newborns are likely to reflect lower energy requests due to the lower neuronal activity [24]. In adults, a linear relationship between subjective response to pain and regional cerebral flow in the contralateral somatosensory brain cortex has been demonstrated [25]. Slater R. et al. [5] supposed that neonates in intensive care process painful experiences at a cortical level, but all the neuronal activity associated with this process increases with postmenstrual age. Nevertheless, this effect is restricted to the contralateral cortex and cannot be applied to changes in global cerebral blood flow related to aging. For this reason, electrophysiological measures, including somatosensory-evoked potentials, in preterm infants, are essential to establish the onset of pain and its related consciousness [5].

Literature data have focused on the timing when a human preterm infant or fetus may start processing pain [3]. Nevertheless, data are sparse and a well-established age of onset in the cortical pain response has not still been well-defined. However, if, on the one hand, it is suggestible that the age of onset may precede the limits of viability (23–24 weeks post menstrual age), on the other hand, we cannot assume that neonatal cortical responses can be directly translatable to the fetal neonate in the uterine environment [3, 5].

The long timing of the cortical responses in the youngest neonates may be attributable to slow synaptic circuits and low conduction velocities within the nociceptive circuitry. This hypothesis is consistent with the long latency reflex responses observed in neonatal age [17]. Despite the long latencies in reflexes responses, these cortical pathways have been identified with a clear onset. In the study of Andrews and Fitzgerald [17], the responses were evident in the two extremely premature infants under morphine at the time of the study. Therefore, morphine seems not to have effects on behavioral and physiological pain scores after heel lance, as shown by Carbajal et al. [26], above all in extremely preterm neonates, even if this topic requires further investigation.

Higher pain processing seems not only taking place within the somatosensory cortex, as adult functional imaging studies have provided a picture of a “pain matrix” within the brain, subdividing this area into a lateral and a medial system, based on the projection areas of the lateral and medial thalamic cortical structures. The somatosensory cortices in the abovementioned lateral system may play a discriminatory role in localizing pain and intensity of painful stimuli, whereas the medial circuits seem to be involved in the anterior cingulated cortex and the insula, and they probably mediate the cognitive-evaluative component of stressful and painful responses [27, 28]. The study shows that the cortical response to pain is more attenuated during sleep, and in adults, this has been interpreted as evidence of cognitive processing of pain [29]. In neonatal age, behavioral states are more undifferentiated for immaturity of neuronal networks and a large proportion of the sleep timing is classified as indeterminate (neither quit, nor active sleep) [30], with a more complex interpretation of the basis of cognitive processing in this age group.

Preterm infants are often exposed to painful procedures in the neonatal intensive care units, as they are exposed to life support interventions. This exposure may explain both immediate and potentially long-term adverse effects, affecting long-term behavior and sensation [31, 32]. However, considering that infants are unable to report their painful sensations directly, indirect behavioral and physiological diagnostic methods are needed to assess pain and its severity [33]. The methods for relevance of pain in the youngest neonates seem to mount a strong and organized response to painful stimuli, even if it is not clear at what level of the central nervous system these responses are sited. Many of these responses can be mediated through spinal cord and deep-brainstem reflex pathways, whereas perception of pain requires cortical processing of noxious stimulation [34].

A prerequisite for the emergence of consciousness is that the thalamocortical and corticocortical connectivity has developed. The aim of our investigation is to temporally analyze the formation of the thalamocortical connections that are the basis of consciousness (Table 2).

**Fig. 4:**
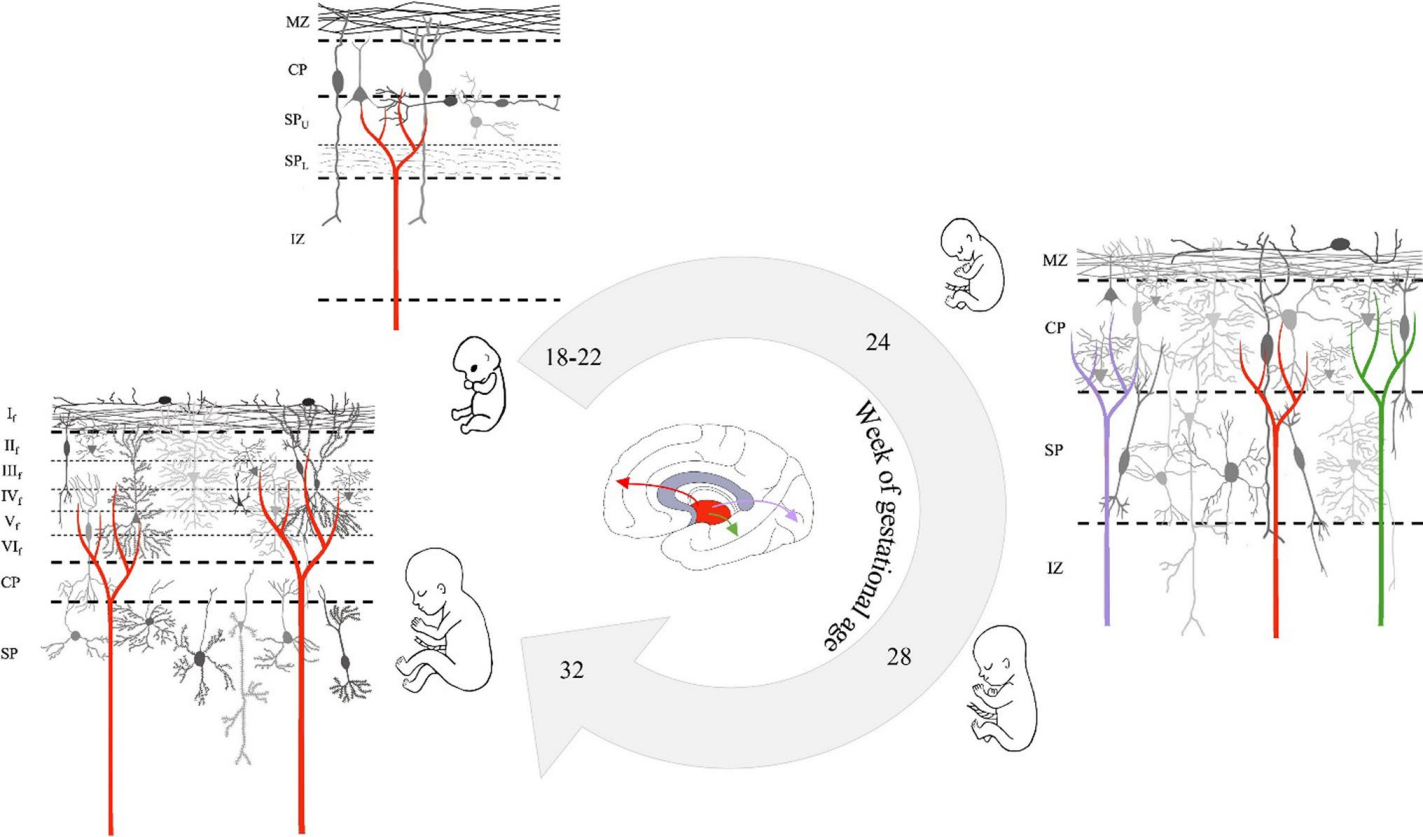
Anatomical development of thalamocortical connectivity and neuronal differentiation of cortex during gestation. At 18–22 weeks of gestation, cortex is organized in marginal zone (MZ), cortical plate (CP), subplate (SP) zone divided in upper (SPU) and lower (SPL) portions, intermediate zone (IZ), and subventricular and ventricular zones. Thalamocortical afferences at this age reach SP zone and stay here until 24 weeks of gestation: SP is considered in fact a “waiting compartment” in which afferents transiently accumulate waiting cortex differentiation. At 24–28 weeks of gestation, thalamic afferents reach CP, also in both visual and auditory cortices, and make synapses with neurons of CP which are to be differentiated. At 32 weeks of gestation, cortex becomes evident in the basic fetal six-layered pattern and thalamic afferents extend along the thickness of the six layers where they connect with mature neurons set with dendritic spines

The neurons from the sensory organs terminate in the subplate of the cortex before the 20–22 weeks of gestational age [6–12]. The subplate serves as a waiting zone and as a guidance hub for the afferents from the thalamus and other areas of the brain. Between 23 and 30 weeks of gestation, there are substantial ingrowths of thalamocortical axons in the cortical plate of the frontal, somatosensory, visual, and auditory cortices, and formation of the first synapses in the deep cortical plate [6–12] (Fig. 4).

During the years, numerous neuroscientific theories of consciousness have been published, including the Recurrent Processing Theory (RPT) [35, 36], the Synchrony Theory (ST) [37], the Integrated Information Theory (IIT) [38], Global Neuronal Workspace Theory (GNWT) [1, 3, 39], the Temporo-spatial Theory of Consciousness (TTC) [40, 41], the Predictive Coding Theory (PCT) [42], the Higher-Order Thought theory (HOT) [43], the Operational Space–time theory (OST) [44], the Entropy Theory of Consciousness [45, 46], the Social Perceptual Attention Theory of Consciousness [47], and the Embodied Theory (ET) [48].

The above theories aim to explain the formation of consciousness in different ways, with different pathways. Diversity is also evident in the fact that each of these theories targets distinct explanada on the side of consistence. In this regard, e.g., phenomenal features like “experience of content,” the “what is like” of phenomenal (P-) consciousness [49], are the main topics of RPT, IIT, ST, and TTC, while theories like GNWT and HOT have targeted more cognitive aspects like the “awareness of content,” or access (A-) consciousness, linked to functions like working memory, access or meta-cognition [50], and top-down attention [47, 51]. Yet, other theories seem to be less specific about what aspect of consciousness is targeted, or in general focus on non-specific mechanisms of perception, such as PCT, or on the association of perceptual states with action, the perception of self-body [52], emotions [53], or the self [54]. Since different aspects of consciousness are focused in these theories, as their explanandum, all these hypotheses may not necessarily be incompatible with each other or exclude one another.

The diversity among all these theories is further amplified by the focus of each of them on different forms of neural activity. Many theories study stimulus-related activity by using various measures as the neural correlate of consciousness. On the other hand, theories like TTC and PCT focus more on pre-stimulus activity or resting state activity (TTC), as this last may strongly influence stimulus-related activity and consciousness itself. Finally, considering that consciousness is already present even in the resting state independently of any stimulation, spontaneous activity, and interactions with incoming information should be taken into account [55, 57].

## Conclusions

This narrative review temporally analyzes the hypothesis on the creation of brain connections at the basis of consciousness. Unfortunately, there is little evidence in the literature. There are no studies that demonstrate the existence of a method useful to study the flow and timing of brain connections in fetal life or in premature babies.

We can conclude that from a neuroanatomical point of view, it is rather unlikely that the infant can be seen as a conscious human before 24 weeks of age, before the thalamocortical connections are established. Further electrophysiological studies are needed on the fetus to confirm this hypothesis.
